# Thyroid-sparing volume-modulated arc therapy in patients with non-distant metastatic nasopharyngeal carcinoma: a feasibility study

**DOI:** 10.3389/fonc.2025.1443226

**Published:** 2025-06-12

**Authors:** Renxian Xie, Jiayang Lu, Qingxin Cai, Longbo Li, Keyan Xie, Tong Chen, Hongxin Huang, Jianzhou Chen, Ying Zhang, Chuangzhen Chen

**Affiliations:** ^1^ Department of Radiation Oncology, Cancer Hospital of Shantou University Medical College, Shantou, China; ^2^ Shantou University Medical College, Shantou, China; ^3^ Department of Radiation Oncology, Jieyang People’s Hospital, Jieyang, China

**Keywords:** nasopharyngeal carcinoma, volume-modulated arc therapy (VMAT), thyroid, hypothyroidism, radiotherapy, dosimetric feasibility

## Abstract

**Purpose:**

To assess the dosimetric feasibility of thyroid-sparing volume-modulated arc therapy (TS VMAT) in patients with non-distant metastatic nasopharyngeal carcinoma.

**Methods:**

TS VMAT plans and non-thyroid-sparing volume-modulated arc therapy (NTS VMAT) plans were created using inverse-planning VMAT and computed tomography datasets of 60 patients from two centers using the Eclipse version 15.6 treatment planning system. These patients were split up into three groups, each consisting of ten patients: the bilateral upper neck irradiation group, the one-side lower neck irradiation group, and the bilateral lower neck irradiation group. Dose volume histograms, the homogeneity index (HI), conformity index (CI), and irradiation doses to the thyroid and other OARs were used to assess the two treatment plans.

**Results:**

There were no statistically significant differences in HI, CI, and dosage distribution to OARs between the two plans, except for the bilateral lower neck irradiation group, where mild but clinically acceptable differences were observed. Surprisingly, the TS VMAT plans significantly reduced the radiation dose to the thyroid gland across all three groups without compromising target coverage, conformity, or dose homogeneity. Specifically, the mean dose to the thyroid was substantially lower in the TS VMAT plans compared to the NTS VMAT plans. Additionally, the volume of the thyroid irradiated with 40 Gy or more was also significantly reduced in the TS VMAT plans.

**Conclusions:**

The TS VMAT plan is appropriate for radiotherapy planning in patients with non-distant metastatic nasopharyngeal carcinoma. The TS VMAT plan reduces radiation dosage to the thyroid gland compared to the NTS VMAT plan, lowering the risk of hypothyroidism without exacerbating the HI, CI, and the irradiation doses to OARs.

## 1 Introduction

Nasopharyngeal carcinoma (NPC) is a malignant tumor of the head and neck originating from epithelial cells of the nasopharynx, which has a high incidence in Southeast Asia, especially in southern China (1, 2). Based on its sensitivity to radiation and chemotherapy, chemoradiotherapy is the main treatment for non-distant metastatic NPC (3). The intensity-modulated radiation therapy (IMRT) technique is routinely used in the treatment of NPC. However, a growing number of studies have found that volume-modulated arc therapy (VMAT) further improves dose conformity, reduces the radiation dose to surrounding normal tissues, and shortens treatment time compared with IMRT (4–6). Based on the fact that 70-80% of patients with NPC have cervical lymph node metastases at the time of initial diagnosis, radiotherapy planning routinely requires the inclusion of bilateral cervical lymph node drainage areas in the target volume (7, 8).

The thyroid gland will inevitably be irradiated during head and neck radiation therapy for NPC because it is located in the anterior neck. There are many metabolic processes and physiological functions of the body that are regulated by the thyroid gland (9, 10). The hormones triiodothyronine (T3) and thyroxine (T4) secreted by the thyroid gland are extremely important for regulating body temperature, metabolism, cholesterol levels, and growth. Exposing the thyroid gland to radiation can potentially result in various disorders, including hypothyroidism, Graves’ disease, thyroiditis, and thyroid cancer (11–13). Hypothyroidism is the most common form of radiation toxicity, which occurs in 40%-50% of patients undergoing radiation therapy to the neck (12). Thyroid hormone imbalances can cause deleterious effects on multiple organ systems. Recent research increasingly substantiates that the incidence of radiation-induced hypothyroidism exhibits a dose-dependent pattern. A higher risk of hypothyroidism has been observed in patients with higher average thyroid radiation doses (12, 14–16), which means that we can reduce the risk of radiotherapy-induced hypothyroidism by minimizing the radiation dose to the thyroid. A study by Lin found that VMAT plans have a clear advantage over IMRT plans in reducing thyroid radiation dose (17).

At present, there is no literature exploring the effects of reducing the radiation dose to the thyroid during the treatment of NPC with VMAT. The aim of this study is to assess the feasibility of thyroid-sparing volume-modulated arc therapy (TS VMAT) for patients with a pathologic diagnosis of NPC. However, it is disappointing that there is no consensus or standardized dose limit for thyroid radiation. A systematic review found that the parameters of thyroid mean dose (Dmean), minimum dose (Dmin), V25, V30, V35, V45, V50, V30-60, VS45, and VS60 were associated with hypothyroidism after radiotherapy (18). Huang et al. recommended that the prescribed dose limits for the thyroid gland should be V25Gy ≤ 60%, V35Gy ≤ 55%, and V45Gy ≤ 45% for the “strict” dose-volume histogram (DVH) line, while V25Gy ≤ 95%, V35Gy ≤ 90%, and V45Gy ≤ 75% should be adopted as the “lenient” DVH line, provided that target coverage is not compromised (19). However, according to previous studies, the thyroid Dmean and the volume receiving at least 40 Gy (V40) can be predictive of hypothyroidism, and limiting them below a certain dose can reduce the incidence of hypothyroidism (12, 20). In clinical practice, it is more widely accepted to limit thyroid Dmean to less than 45 Gy (12, 15) and V40 to less than 85% (21). Therefore, in this study, thyroid-sparing was defined as the thyroid Dmean of no more than 45 Gy and V40 of no more than 85%.

## 2 Materials and methods

### 2.1 Ethics statement

Our study protocol received approval from the Ethical Commission of the Cancer Hospital of Shantou University Medical College. As this study did not involve treatment interventions, the institutional review board determined that informed consent from the participants was unnecessary and waived that requirement. To protect patients’ confidentiality, the patient information was anonymized and de-identified.

### 2.2 Patient characteristics

This dosimetric feasibility study included 60 patients diagnosed with non-distant metastatic NPC who received radiotherapy at the Affiliated Cancer Hospital of Shantou University Medical College (A center) and Jieyang People’s Hospital (B center). These patients received bilateral upper neck irradiation (the Bilateral UNI group), bilateral upper neck irradiation and one-side of lower neck irradiation (the One-side LNI group), or bilateral upper and lower neck irradiation (the Bilateral LNI group) between January 1st, 2020, and March 1st, 2023, and were evenly distributed into three groups. In each group, the patient number from the two hospitals was the same. In this study, patient inclusion was not determined by a dosimetrist, but was carefully screened by experienced clinicians. **Table 1** lists the characteristics of these patients, including age, gender, T stage, N stage, AJCC TNM stage, thyroid volume, planning target volume of nasopharynx (PTVnx), planning target volume of metastatic nodes (PTVnd), planning target volume receiving 60Gy (PTV60), and planning target volume receiving 54Gy (PTV54).

**Table 1:** Characteristics of patients with non-distant metastatic nasopharyngeal carcinoma.

	Bilateral upper neck irradiation group (n = 20)	One-side lower neck irradiation group (n = 20)	Bilateral lower neck irradiation group (n = 20)	P-value
**age**, Mean ± SD	57.7 ± 13.3	55.5 ± 10.9	52.3 ± 12.6	0.384
gender				0.045*
F	2	5	9	
M	18	15	11	
T stage				0.640
1	8	4	3	
2	2	4	7	
3	8	10	8	
4	2	2	2	
N stage				<0.001*
0	20	0	0	
1	0	19	0	
2	0	0	16	
3	0	1	4	
AJCC 8th TNM stage				0.207
I	4	4	0	
II	5	1	3	
III	8	11	13	
IVA	3	4	4	
Thyroid volume(cm^3^), Mean ± SD	17.6 ± 6.5	20.3 ± 26.8	16.8 ± 9.7	0.789
PTVnx volume(cm^3^), Mean ± SD	71.1 ± 36	66.7 ± 39.9	76.4 ± 53.3	0.780
PTVnd volume(cm^3^), Mean ± SD	–	31.7 ± 17	64.2 ± 26.4	<0.001*
PTV60 volume(cm^3^), Mean ± SD	148.7 ± 65.9	145.5 ± 83.7	170.1 ± 86.6	0.569
PTV54 volume(cm^3^), Mean ± SD	657.3 ± 145.8	655.7 ± 125.9	764.6 ± 118.8	0.015*

T, Tumor; N, regional lymph node; TNM, Tumor Node Metastasis; AJCC, American Joint Committee on Cancer; PTVnx, Planning Target Volume of nasopharynx; PTVnd, Planning Target Volume of the metastatic lymph nodes; PTV60, Planning Target Volume receiving 60 Gy; PTV54, Planning Target Volume receiving 54 Gy; M, Male; F, Female; SD, Standard Deviation.

* for P-value ≤ 0.05.

### 2.3 CT simulation

All patients were positioned in a supine posture and securely immobilized using a tailor-made thermoplastic mask that spanned from the cranium to the upper thoracic region. Computed tomography (CT) scans, employing intravenous contrast, were conducted with a 3 mm slice thickness, encompassing the cranial region down to a distance of 2 cm below the sternoclavicular joint. The scans were conducted using a Philips Brilliance CT Big Bore Oncology Configuration 16-slice CT scanner (Cleveland, OH, USA). Subsequently, the CT images were exported to the Eclipse treatment planning system, version 15.6 (Varian Medical System, Inc., Palo Alto, CA, USA) for the purpose of precisely delineating the target areas and organs-at-risk (OARs), as well as formulating the treatment plans.

### 2.4 Target delineation and OAR definition

All target volumes and OARs were delineated by our radiation oncologists according to the Guidelines of the Chinese Society of Clinical Oncology (CSCO, version 2023) and the Chinese Guidelines for Radiation Therapy of Nasopharyngeal Carcinoma (version 2022). The target volumes and OARs were precisely localized and delineated based on the CT images and the pre-treatment MRI images in fusion. The gross tumor volume in the nasopharynx (GTVnx), which includes all visible primary tumor mass and retropharyngeal lymph node involvement, was outlined through a thorough evaluation using CT, MRI scans, and endoscopic observations. Enlarged positive lymph nodes in the neck were identified as the gross tumor volume of the metastatic nodes (GTVnd). CTV60, the clinical target volume for high-risk areas, included the GTVnx and an extra 5 mm of the surrounding subclinical lesion. CTV54 was defined as the low-risk clinical target volume requiring preventive irradiation. It included GTVnx, GTVnd, the whole nasopharynx, retropharyngeal nodal areas, and important nearby structures like the skull base, sphenoid sinus, pterygoid fossae, parapharyngeal space, and clivus. The extension also included the rear section of the nasal cavity, the maxillary sinuses, a segment of the posterior ethmoid sinus, and the cervical nodal regions that received elective prophylactic irradiation. The PTVnx, PTVnd, PTV60, and PTV54 were created by extending 5 mm beyond the edges of the GTVnx, GTVnd, CTV60, and CTV54, respectively.

When assessing the dose homogeneity index (HI) for both PTV60 and PTV54, we excluded higher doses from PTVnx and PTVnd. In order to achieve this, PTV60minus was calculated by subtracting 3 mm expansion volumes from both PTVnx and PTVnd from the PTV60. Similarly, PTV54minus was established by subtracting 3 mm expansion volumes of PTVnx, PTVnd, and PTV60 from the PTV54. Using the CT images, we meticulously outlined the OARs, including the spinal cord, brainstem, lenses, optic nerves, optic chiasm, larynx, oral cavity, parotid glands, eyeballs, and pituitary gland. To further safeguard critical structures, organ-at-risk volumes for the spinal cord (spinal cord PRV) and the brainstem (brainstem PRV) were delineated by adding 5 mm and 3 mm margins to the spinal cord and brainstem, respectively. Normal tissues were defined as the body volumes that did not consist of PTVs.

We identified the thyroid as an organ at risk in TS VMAT plans during this study, unlike the NTS VMAT plans, which did not limit the thyroid dose. A radiotherapist with at least 5 years of clinical experience will modify the plan if PTVs involve OARs.

### 2.5 VMAT planning

In this study, the VMAT technique was used with a 6-MV photon beam from a TrueBeam (Varian Medical System, Inc., Pao Alto, CA) linear accelerator. The TS VMAT plans and NTS VMAT plans were created using the inverse-planning VMAT with Eclipse version 15.6 (Varian Medical Systems, Palo Alto, CA, USA) treatment planning system.

Three coplanar full arcs were used for the planning. The collimator angles were 10-30°/350-330°for the first two arcs and 0°for the third arc, aiming to maximize the modulation of the multi-leaf collimator (MLC). To create controlled dose gradients around the PTVs, dose-limiting ring structures were deployed. Optimization of treatment plans was conducted using the Photon Optimizer (PO, version 15.6.06) algorithm. For the purposes of final dose calculations, the Anisotropic Analytical Algorithm (AAA, version 15.6.06) was utilized with a grid size of 2.5 mm.

The VMAT plans implemented in this study adhered to the following prescribed dose regimens for the patients: 70.4 Gy (32 fractions, with 2.2 Gy per fraction) for PTVnx, 68 Gy (32 fractions, with 2.12 Gy per fraction) for PTVnd, 60 Gy (32 fractions, with 1.88 Gy per fraction) for PTV60, and 54 Gy (32 fractions, with 1.68 Gy per fraction) for PTV54. Each treatment plan was meticulously normalized to guarantee that more than 95% of each PTV volume received the prescribed dose.

### 2.6 Plan evaluation

In order for a plan to be eligible, it must minimize radiation doses to the thyroid and other OARs while guaranteeing that the PTV receives at least 95% and no more than 110% of the recommended dosage. The achievement of TS VMAT programs was assessed by making sure that the thyroid V40 and Dmean did not surpass 85% and 45 Gy, respectively. Sufficient thyroid sparing was never attained at the expense of adequate PTV coverage. Priority over thyroid limits was given to dose restrictions to other OARs (such as the brainstem and spinal cord) when clinically necessary.

The treatment plans were assessed using various parameters, including the dose volume histogram (DVH), HI, conformity index (CI), and specific dose values such as D98 (dose to 98% volume), D50 (dose to 50% volume), and D2 (dose to 2% volume) for PTVnx, PTVnd, PTV60_minus_, and PTV54_minus_.

The HI (22) was used to assess the target dose homogeneity, and it was defined using the following formula:


(1) $$HI=\frac{D2-D98}{D50}$$

The CI was used to assess the target dose conformity and was defined by the formula:


(2) $$CI=\frac{TV_{RI}}{TV}\times \frac{TV_{RI}}{V_{RI}}$$

To assess hot and cold spots, D2 and D98 were selected as the nearest maximal and minimal doses for the PTVs. TV_RI_ is the target volume (TV) covered by the prescription dose, and V_RI_ is the total body volume receiving the prescription dose. Ideally, the HI value should be 0, suggesting better homogeneity with lower values, whereas the CI value should ideally be 1, indicating better conformality with higher values.

We also investigated the metrics that assess the radiation dose to the thyroid, such as Dmax, Dmean, Dmin, V25, V30, V35, V40, V45, V50, V30-60, VS45, and VS60. The planning restrictions for the OARs are as follows. Dmax<60Gy in brainstem PRV, Dmax<54Gy in brainstem, Dmax<50 Gy in spinal cord PRV, Dmax<45Gy in spinal cord, Dmax<9Gy in lens, Dmax<54Gy in optic nerves and optic chiasm, Dmean<40Gy in parotids, oral cavity, and larynx, Dmax<50Gy in eyeballs, and Dmean<50Gy in pituitary. If one of the above OARs exceeds the limit, our skilled physicians will weigh the radiation dose to the radiotherapy target area against the radiation dose to the OARs and make the necessary changes.

A penalty score was constructed based on the mean absolute dose deviation (MADD), which quantifies the extent to which the planned dose diverges from a specified reference dose for a given structure (23). The penalty score associated with a patient’s treatment plan is defined as a weighted sum of the MADDs. In contrast to the homogeneity index, which is a dimensionless ratio applicable solely to target volumes, the defined penalty score can be interpreted as a weighted average of all dose deviations for OAR and PTV (24). In this study, the wi weights initially selected for the One-side LNI group and the Bilateral LNI group were determined as follows: 0.12 for PTVnx, 0.10 for PTVnd, 0.07 for PTV60HI, 0.06 for PTV54HI, and 0.076, 0.084, 0.025, 0.025, 0.02, 0.02, 0.02, 0.09, 0.04, 0.04, 0.02, 0.015, 0.015, 0.01, 0.066, 0.074, and 0.01 for the brainstem PRV, brainstem, left eye, right eye, larynx, left len, right len, optic chiasm, left optic nerve, right optic nerve, oral cavity, left parotid, right parotid, pituitary, spinal cord PRV, spinal cord, and thyroid, respectively. And the wi weights initially selected by the Bilateral UNI group were determined as follows: 0.13 for PTVnx, 0.077 for PTV60HI, 0.067 for PTV54HI, 0.079 for brainstem PRV, 0.088 for brainstem, 0.029 for left eye, 0.029 for right eye, 0.027 for larynx, 0.024 for left len, 0.024 for right len, 0.097 for optic chiasm, 0.044 for left optic nerve, 0.044 for right optic nerve, 0.027 for oral cavity, 0.019 for left parotid, 0.019 for right parotid, 0.017 for pituitary, 0.070 for spinal cord PRV, 0.077 for spinal cord, and 0.017 for thyroid, respectively. A set of ordinal priorities was established assigning (brainstem+brainstem PRV) > (spinal cord+spinal cord PRV) > PTVnx > PTVnd > optic chiasm > optic nerve > PTV60HI > PTV54HI > eye > len > parotid > oral cavity > pituitary > larynx > thyroid, which were then converted to the numerical weights. The above settings are based on clinical experience and previous literature (25).

### 2.7 Statistical analysis

We used IBM SPSS Statistics (version R26.0.0.2) and Free Statistics software versions 1.7 for statistical analysis. The data are presented in the format of “mean ± SD,” where SD represents the standard deviation. Paired samples with a normal distribution were compared using the paired t-test, while paired samples without a normal distribution were analyzed using the Wilcoxon Signed Rank test. A p-value of less than 0.05 was the criterion for statistical significance.

## 3 Results

### 3.1 Target coverage, conformity and dose homogeneity

**Figure 1:**
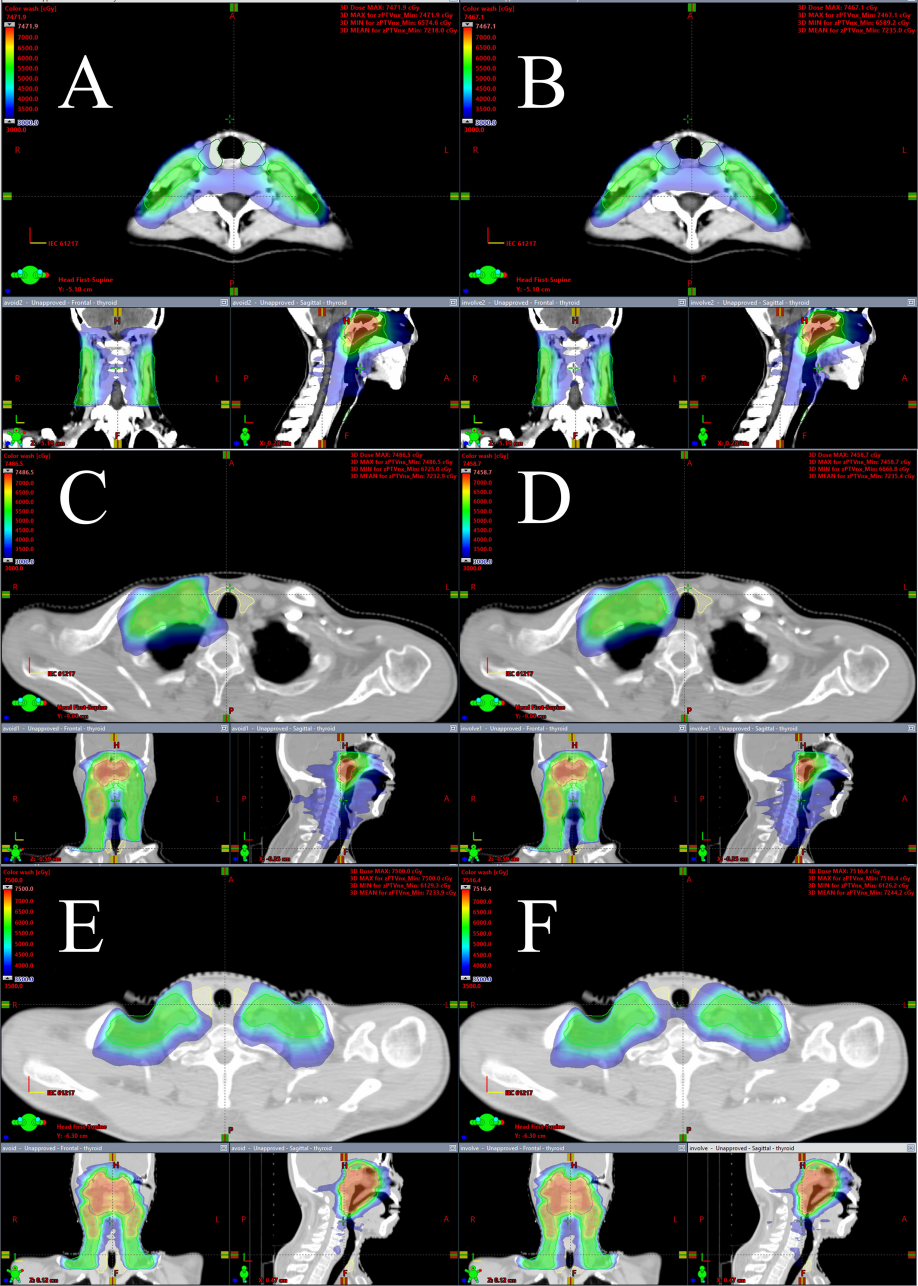
**(A, B)** Dose distributions on three axial views of one nasopharyngeal carcinoma case in the Bilateral UNI group for TS VMAT plan and NTS VMAT plan, **(C, D)** Dose distributions on three axial views of one nasopharyngeal carcinoma case in the One-side LNI group for TS VMAT plan and NTS VMAT plan, **(E, F)** Dose distributions on three axial views of one nasopharyngeal carcinoma case in the Bilateral LNI group for TS VMAT plan and NTS VMAT plan.

**Figure 2:**
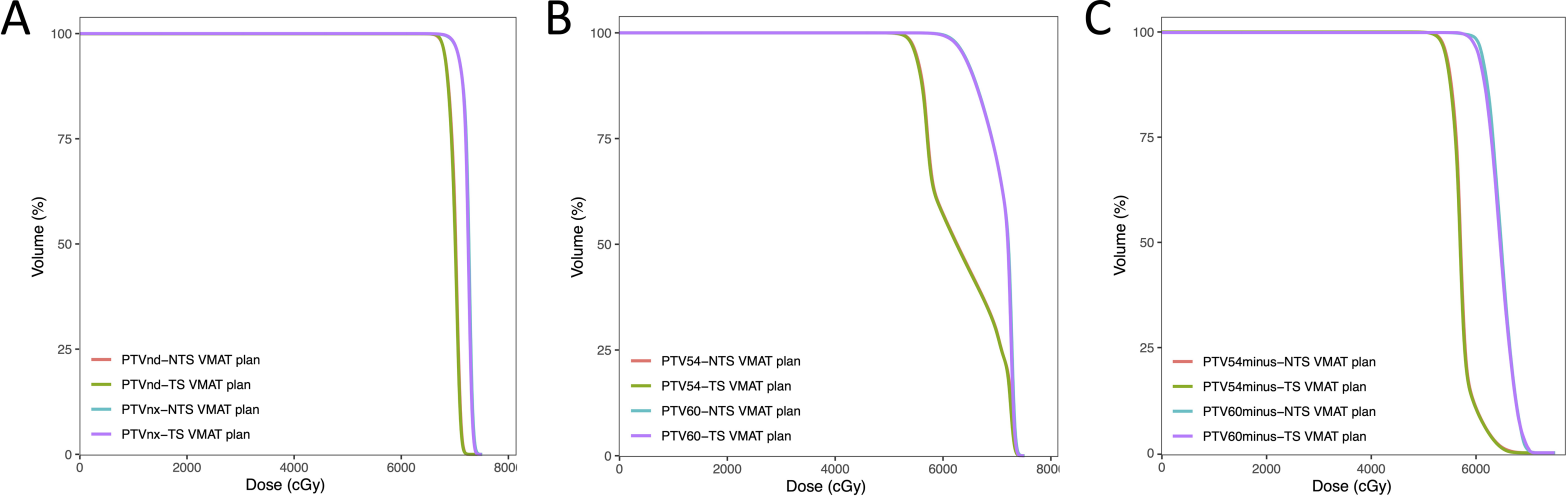
Dose-volume histograms (DVHs) comparison of all PTVs for one nasopharyngeal carcinoma case in the Bilateral LNI group for NTS VMAT plans and NTS VMAT plans.

All of the TS VMAT plans and NTS VMAT plans were clinically acceptable. **Figure 1** displays the dose distributions for the NTS VMAT plans and TS VMAT plans for one patient in each of the three groups. **Table 2** shows the dosimetric results for PTVnx, PTVnd, PTV60, and PTV54 of all plans. For patients who received bilateral upper neck or one-side lower neck irradiation, there were no significant differences between TS VMAT plans and NTS VMAT plans in terms of HI and CI for all PTVs, indicating that TS VMAT plans ensured no significant dosage distribution change for target volume. However, we observed slight fluctuations in TS VMAT plans in the Bilateral LNI group, such as HI for PTVnd, CI for PTV60, CI for PTV54, and HI for PTV54_minus_. It is worth noting that all of these fluctuations were clinically acceptable. The DVH of all PTVs for one patient in the Bilateral LNI group is shown in **Figure 2**. These results indicated that the TS VMAT plans were not inferior to the traditional NTS VMAT plans in terms of dose distribution.

**Table 2:** Dosage distribution in PTVnx, PTVnd, PTV60_minus_ and PTV54_minus_ in NTS VMAT plans and TS VMAT plans.

	Bilateral upper neck irradiation group			One-side lower neck irradiation group			Bilateral lower neck irradiation group		
	NTS VMAT	TS VMAT	P-value	NTS VMAT	TS VMAT	P-value	NTS VMAT	TS VMAT	P-value
	(Mean ± SD)	(Mean ± SD)		(Mean ± SD)	(Mean ± SD)		(Mean ± SD)	(Mean ± SD)	
PTVnx									
D98 (Gy)	69.91 ± 0.26	69.92 ± 0.26	0.611	69.88 ± 0.33	69.82 ± 0.41	0.370	69.93 ± 0.35	69.94 ± 0.35	0.588
D50 (Gy)	72.33 ± 0.30	72.29 ± 0.32	0.166	72.33 ± 0.44	72.33 ± 0.46	0.879	72.53 ± 0.34	72.54 ± 0.37	0.768
D2 (Gy)	73.48 ± 0.40	73.51 ± 0.48	0.911	73.58 ± 0.59	73.59 ± 0.56	0.638	73.84 ± 0.46	73.87 ± 0.49	0.242
HI	0.05 ± 0.01	0.05 ± 0.01	0.970	0.05 ± 0.01	0.05 ± 0.01	0.455	0.05 ± 0.01	0.05 ± 0.01	0.366
CI	0.87 ± 0.04	0.87 ± 0.03	0.352	0.66 ± 0.17	0.66 ± 0.17	0.852	0.56 ± 0.15	0.56 ± 0.15	0.809
PTVnd									
D98 (Gy)	–	–	–	67.84 ± 0.75	67.97 ± 0.45	0.896	67.71 ± 0.34	67.66 ± 0.35	0.024*
D50 (Gy)	–	–	–	70.39 ± 0.41	70.38 ± 0.37	0.739	70.65 ± 0.79	70.56 ± 0.45	1.000
D2 (Gy)	–	–	–	71.92 ± 0.59	71.89 ± 0.66	0.638	72.37 ± 0.78	72.42 ± 0.84	0.263
HI	–	–	–	0.06 ± 0.01	0.06 ± 0.01	0.232	0.07 ± 0.01	0.07 ± 0.01	0.049*
CI	–	–	–	0.26 ± 0.17	0.26 ± 0.17	0.502	0.36 ± 0.13	0.36 ± 0.13	0.126
PTV60minus									
D98 (Gy)	60.27 ± 0.74	60.29 ± 0.76	0.730	60.26 ± 0.63	60.17 ± 0.64	0.344	60.42 ± 0.53	60.40 ± 0.64	0.573
D50 (Gy)	64.73 ± 1.47	64.71 ± 1.49	0.671	64.42 ± 1.33	64.36 ± 1.30	0.177	64.26 ± 1.52	64.26 ± 1.50	0.814
D2 (Gy)	68.84 ± 1.90	68.77 ± 1.89	0.358	68.72 ± 1.59	68.67 ± 1.54	0.540	68.20 ± 1.97	68.34 ± 2.09	0.052
HI	0.13 ± 0.03	0.13 ± 0.03	0.291	0.13 ± 0.02	0.13 ± 0.02	0.664	0.12 ± 0.03	0.12 ± 0.03	0.115
PTV60									
CI	0.76 ± 0.09	0.76 ± 0.09	0.706	0.51 ± 0.14	0.51 ± 0.14	0.650	0.44 ± 0.15	0.43 ± 0.15	0.015*
PTV54minus									
D98 (Gy)	52.80 ± 0.60	52.68 ± 0.83	0.247	52.83 ± 0.61	52.71 ± 0.79	0.204	52.76 ± 0.54	52.10 ± 0.90	0.000*
D50 (Gy)	57.03 ± 0.37	57.01 ± 0.37	0.474	57.12 ± 0.30	57.12 ± 0.29	0.681	57.41 ± 0.50	57.47 ± 0.55	0.018*
D2 (Gy)	59.69 ± 0.89	59.71 ± 0.93	0.709	63.24 ± 1.21	63.13 ± 1.25	0.156	63.98 ± 1.15	64.05 ± 1.01	0.311
HI	0.12 ± 0.02	0.12 ± 0.02	0.296	0.18 ± 0.02	0.18 ± 0.03	1.000	0.20 ± 0.02	0.21 ± 0.02	0.001*
PTV54									
CI	0.89 ± 0.02	0.89 ± 0.03	0.911	0.87 ± 0.03	0.87 ± 0.03	0.840	0.87 ± 0.03	0.86 ± 0.03	0.002*

NTS VMAT, non-thyroid-sparing volume modulated arc therapy; TS VMAT, thyroid-sparing volume modulated arc therapy; PTVnx, Planning Target Volume of nasopharynx; PTVnd, Planning Target Volume of the metastatic lymph nodes; PTV60, Planning Target Volume receiving 60 Gy; PTV60_minus_, PTV60 minus PTVnx and PTVnd 3 mm expansion volume; PTV54, Planning Target Volume receiving 54 Gy; PTV54_minus_, PTV54 minus PTV60; PTVnx and PTVnd 3 mm expansion volume; D98, dose to 98% volume; D50, dose to 50% volume; D2, dose to 2% volume; HI, homogeneity index; CI, conformity index; *: P<0.05; SD, Standard Deviation.

When analyzed separately, data from the A center revealed no significant differences in HI and CI for all PTVs between the TS VMAT plans and the NTS VMAT plans for all groups. As for data from the B center, slight changes were observed during the execution of the TS VMAT plans, such as HI for PTVnd, CI for PTV60, CI for PTV54, and HI for PTV54minus in the Bilateral LNI group, which was similar to the results of the bicenter data analysis. The **Supplementary Material** includes details on the DVH for all PTVs in the One-side LNI and Bilateral UNI groups, as well as an analysis of target coverage, conformity, and dose homogeneity for the two plans at the single center.

### 3.2 OARs

**Figure 3:**
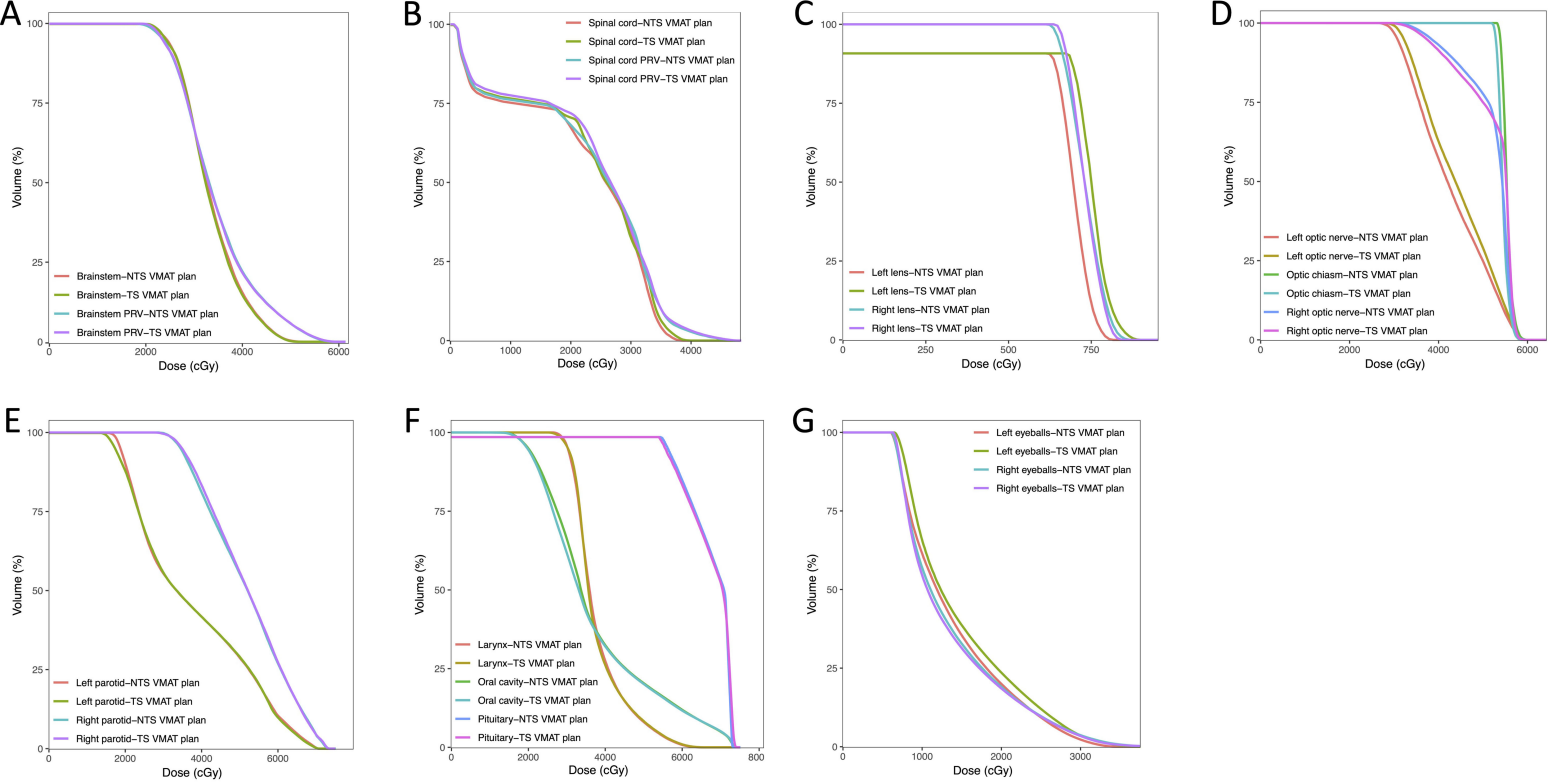
Dose-volume histograms (DVHs) comparison of OARs for one nasopharyngeal carcinoma case in the Bilateral LNI group for NTS VMAT plans and NTS VMAT plans.

The dosimetric results for OARs in all plans are shown in **Table 3**. Radiation doses to most OARs were not significantly different between the NTS VMAT program and the TS VMAT program in all three groups. In the Bilateral LNI group, after limiting thyroid irradiation, there were slight increases in dosage distribution in the spinal cord PRV (40.02 ± 1.93 vs 40.58 ± 2.10, p = 0.001), left optic nerve (37.86 ± 17.91 vs 38.29 ± 17.91, p = 0.018), and right parotid gland (32.66 ± 5.53 vs 32.78 ± 5.54, p = 0.033). These increases, although statistically significant, remained clinically acceptable. The DVH of the OARs for one patient in the Bilateral LNI group is shown in **Figure 3**.

**Table 3:** Dosage distribution in OARs in NTS VMAT plans and TS VMAT plans.

	Bilateral upper neck irradiation group			One-side lower neck irradiation group			Bilateral lower neck irradiation group		
	NTS VMAT	TS VMAT	P-value	NTS VMAT	TS VMAT	P-value	NTS VMAT	TS VMAT	P-value
	(Mean ± SD)	(Mean ± SD)		(Mean ± SD)	(Mean ± SD)		(Mean ± SD)	(Mean ± SD)	
Brainstem PRV									
Dmax (Gy)	56.73 ± 5.09	56.57 ± 5.30	0.254	58.11 ± 3.22	58.01 ± 3.18	0.450	57.25 ± 4.15	57.13 ± 4.32	0.173
Brainstem									
Dmax (Gy)	49.05 ± 6.34	49.02 ± 6.20	0.798	50.04 ± 4.84	50.18 ± 5.07	0.601	49.71 ± 5.29	49.62 ± 5.14	0.505
Spinal cord PRV									
Dmax (Gy)	46.47 ± 3.79	46.03 ± 2.91	0.881	46.22 ± 2.40	46.46 ± 1.93	0.681	47.10 ± 2.07	47.26 ± 2.48	0.086
Spinal cord									
Dmax (Gy)	38.99 ± 2.31	39.21 ± 2.27	0.478	39.42 ± 1.55	39.32 ± 1.68	0.471	40.02 ± 1.93	40.58 ± 2.10	0.001*
Left lens									
Dmax (Gy)	6.19 ± 1.51	6.06 ± 1.47	0.071	5.77 ± 1.49	5.71 ± 1.54	0.342	6.48 ± 1.45	6.63 ± 1.71	0.411
Right lens									
Dmax (Gy)	6.10 ± 1.51	6.06 ± 1.47	0.343	5.78 ± 1.76	5.77 ± 1.71	0.846	6.36 ± 1.59	6.38 ± 1.68	0.746
Left optic nerves									
Dmax (Gy)	37.24 ± 17.48	37.22 ± 17.09	0.126	28.31 ± 17.84	28.83 ± 17.81	0.179	37.86 ± 17.91	38.29 ± 17.91	0.018*
Right optic nerves									
Dmax (Gy)	33.90 ± 15.24	33.52 ± 14.88	0.272	29.79 ± 15.91	29.59 ± 16.07	0.455	37.13 ± 16.88	37.39 ± 16.78	0.340
optic chiasm									
Dmax (Gy)	37.11 ± 18.44	36.89 ± 18.43	0.370	34.42 ± 18.25	34.25 ± 18.34	0.940	38.77 ± 18.82	37.65 ± 19.70	0.156
Left parotids									
Dmean (Gy)	30.24 ± 2.28	30.14 ± 2.26	0.069	32.22 ± 3.71	32.17 ± 3.70	0.135	30.00 ± 7.61	31.60 ± 3.14	0.723
Right parotids									
Dmean (Gy)	30.26 ± 2.71	30.22 ± 2.70	0.562	31.27 ± 3.18	31.31 ± 3.12	0.581	32.66 ± 5.53	32.78 ± 5.54	0.033*
oral cavity									
Dmean (Gy)	33.67 ± 1.62	33.65 ± 1.73	0.794	35.47 ± 3.47	35.37 ± 3.48	0.296	36.39 ± 4.11	36.40 ± 4.05	0.955
larynx									
Dmean (Gy)	34.37 ± 1.72	34.30 ± 1.73	0.023*	35.30 ± 2.36	35.37 ± 3.48	0.351	36.29 ± 3.71	36.32 ± 3.74	0.627
Left eyeballs									
Dmax (Gy)	18.56 ± 8.95	17.69 ± 8.06	0.079	17.31 ± 7.03	17.30 ± 6.86	0.370	20.48 ± 9.37	20.47 ± 9.44	0.972
Right eyeballs									
Dmax (Gy)	18.58 ± 7.10	18.66 ± 6.98	0.835	18.95 ± 8.75	18.88 ± 8.68	0.861	21.75 ± 12.50	21.83 ± 12.54	0.799
pituitary									
Dmean (Gy)	44.92 ± 15.68	45.20 ± 15.41	0.223	46.83 ± 15.52	47.87 ± 15.08	0.093	47.57 ± 17.64	47.87 ± 17.61	0.837

NTS VMAT, non-thyroid-sparing volume modulated arc therapy; TS VMAT, thyroid-sparing volume modulated arc therapy; PRV, planning organs-at-risk volume; Dmax, maximum dose; Dmean, mean dose, *: P<0.05, SD, Standard Deviation.

In Center A, the variations in dosage distribution introduced to the OARs by the TS VMAT plans were not pronounced, with only a slight increase observed in the spinal cord for the Bilateral LNI group. Conversely, in Center B, there was a modest increase in radiation dose to the spinal cord, left and right parotid glands, and oral cavity in the TS VMAT plans for the Bilateral LNI group. The **Supplementary Material** provides further details on the DVH of the OARs within the One-side LNI group and the Bilateral UNI group, as well as an analysis of the OARs for both treatment plans conducted at the single center.

### 3.3 Thyroid

**Figure 4:**
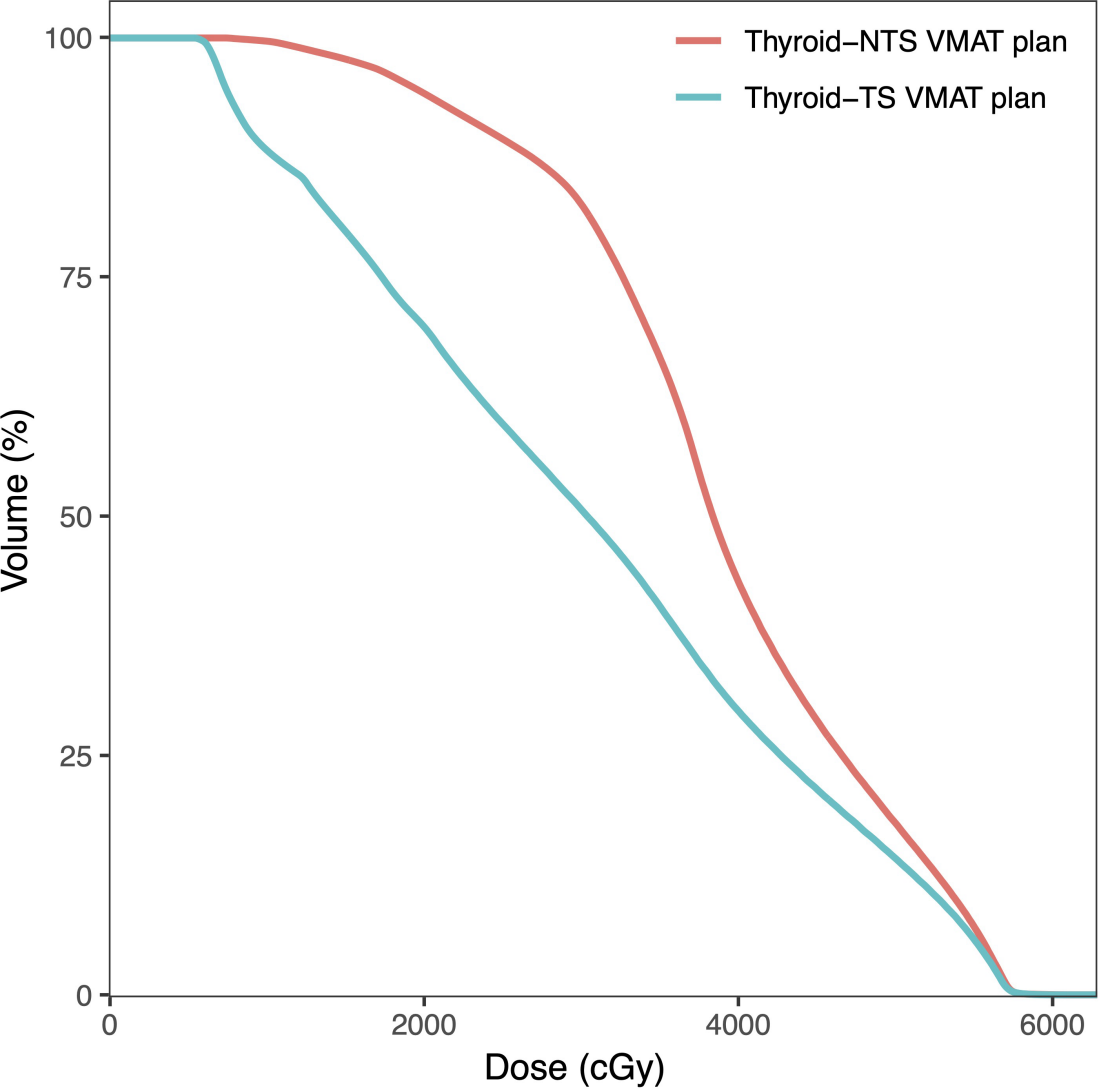
Dose-volume histograms (DVHs) comparison of thyroid for one nasopharyngeal carcinoma case in the Bilateral LNI group for NTS VMAT plans and NTS VMAT plans.

We conducted an analysis of the cumulative dose received by the thyroid under two VMAT plans. **Table 4** lists the detailed values. Thyroid Dmean, Dmin, V25, V30, V35, V40, V45, V50, V30-60, and VS45 for TS VMAT plans were significantly lower in contrast to NTS VMAT plans for all groups. For thyroid Dmax, there were significantly lower in the TS VMAT plans only in the Bilateral LNI group (p = 0.048), and the thyroid Dmax results were similar in the other two groups with no significant differences. The DVH of the thyroid for one patient in the Bilateral LNI group is shown in **Figure 4**. In conclusion, TS-VMAT plans could be crucial in preventing excessive radiation dose to the thyroid when compared with traditional NTS-VMAT plans.

**Table 4:** Dosage distribution in the thyroid in NTS VMAT plans and TS VMAT plans.

	Bilateral upper neck irradiation group			One-side lower neck irradiation group			Bilateral lower neck irradiation group		
	NTS VMAT	TS VMAT	P-value	NTS VMAT	TS VMAT	P-value	NTS VMAT	TS VMAT	P-value
	(Mean ± SD)	(Mean ± SD)		(Mean ± SD)	(Mean ± SD)		(Mean ± SD)	(Mean ± SD)	
Thyroid									
Dmin (Gy)	5.26 ± 2.08	4.15 ± 1.26	0.000*	19.14 ± 7.67	7.66 ± 2.13	0.000*	40.02 ± 1.93	12.22 ± 2.25	<0.001*
Dmean (Gy)	29.67 ± 7.19	20.56 ± 4.12	0.000*	39.86 ± 4.35	26.18 ± 4.74	0.000*	47.35 ± 2.57	34.17 ± 6.10	<0.001*
Dmax (Gy)	57.93 ± 1.32	57.02 ± 2.60	0.100	59.46 ± 3.22	59.73 ± 3.68	0.205	60.31 ± 3.78	59.13 ± 4.61	0.048*
V25 (%)	58.03 ± 18.17	35.31 ± 14.02	<0.001*	86.41 ± 13.56	44.60 ± 11.25	<0.001*	99.41 ± 2.36	67.28 ± 13.33	<0.001*
V30 (%)	51.83 ± 17.70	30.09 ± 12.42	<0.001*	78.87 ± 16.39	37.26 ± 12.44	<0.001*	98.98 ± 3.87	57.23 ± 15.75	<0.001*
V35 (%)	44.91 ± 16.73	24.85 ± 10.67	<0.001*	68.94 ± 16.47	30.77 ± 13.59	<0.001*	96.41 ± 7.55	47.65 ± 17.83	<0.001*
V40 (%)	51.71 ± 71.82	18.00 ± 6.82	0.000*	53.88 ± 13.95	24.71 ± 13.74	0.000*	85.53 ± 12.94	37.74 ± 19.51	<0.001*
V45 (%)	26.22 ± 15.42	13.14 ± 6.17	<0.001*	37.23 ± 12.48	18.70 ± 12.92	<0.001*	64.66 ± 13.12	27.49 ± 19.59	<0.001*
VS45 (cc)	4.88 ± 5.13	2.35 ± 1.53	<0.001*	5.25 ± 2.17	2.70 ± 2.03	<0.001*	9.90 ± 3.29	4.45 ± 3.62	<0.001*
V50 (%)	15.80 ± 10.47	7.31 ± 4.78	<0.001*	22.67 ± 10.33	12.45 ± 11.01	<0.001*	40.29 ± 11.61	17.72 ± 16.65	<0.001*
V30-60 (%)	51.82 ± 17.68	30.09 ± 12.42	<0.001*	78.23 ± 15.80	36.90 ± 12.23	<0.001*	98.71 ± 3.90	57.12 ± 15.63	<0.001*
VS60 (cc)	0.00 ± 0.00	0.00 ± 0.00	0.317	0.07 ± 0.25	0.04 ± 0.14	0.273	0.04 ± 0.11	0.01 ± 0.06	0.068

NTS VMAT, non-thyroid-sparing volume modulated arc therapy; TS VMAT, thyroid-sparing volume modulated arc therapy; Dmax, maximum dose; Dmean, mean dose; Dmin, minimum dose; Vx, percentage of the volume irradiated with x Gy or more, VSx: the volume irradiated with x Gy or more, *: P<0.05, SD, Standard Deviation.

Unexpectedly, even analyses conducted at a single center demonstrated significant reductions in thyroid Dmean, Dmin, V25, V30, V35, V40, V45, V50, V30-60, and VS45 with the TS VMAT plans compared to the NTS VMAT plans. However, at Center A, no significant difference in thyroid Dmax was observed between the Bilateral UNI group and the Bilateral LNI group. Similarly, at Center B, the thyroid Dmax for the One-side LNI group and the Bilateral LNI group did not exhibit significant differences. The **Supplementary Material** provides further details on the DVH of the thyroid within the One-side LNI group and the Bilateral UNI group, as well as an analysis of the thyroid for both treatment plans conducted at the single center.

### 3.4 Mean absolute dose deviation

The MADD values for all structures were compared between TS VMAT and NTS VMAT plans across three irradiation groups (**Table 5**). In the Bilateral UNI group, no significant differences were observed in MADD for all structures, including PTVnx, PTV60, PTV54, and OARs such as the brainstem PRV. Similarly, in the One-side LNI group, MADD values for all PTVs and OARs remained comparable between the two plans. Notably, in the Bilateral LNI group, TS VMAT plans demonstrated mild but statistically significant increases in MADD for the spinal cord PRV (28.65 ± 4.39 vs. 27.29 ± 3.92 Gy, p=0.040). However, these differences were clinically acceptable and did not compromise OAR constraints. Importantly, the TS VMAT plans significantly reduced MADD of the thyroid in all groups of patients, although only statistically significant in the One-side LNI group.

**Table 5:** Mean Absolute Dose Deviation (MADD) for all structures in TS VMAT plans and NTS VMAT plans.

	Bilateral upper neck irradiation group			One-side lower neck irradiation group			Bilateral lower neck irradiation group		
	NTS VMAT	TS VMAT	P-value	NTS VMAT	TS VMAT	P-value	NTS VMAT	TS VMAT	P-value
	(Mean ± SD)	(Mean ± SD)		(Mean ± SD)	(Mean ± SD)		(Mean ± SD)	(Mean ± SD)	
PTVnx	1.79 ± 0.31	1.79 ± 0.31	0.433	3.11 ± 0.32	3.11 ± 0.31	0.779	2.03 ± 0.31	2.05 ± 0.34	0.485
PTVnd	/	/	/	3.39 ± 1.94	3.43 ± 1.93	0.463	2.44 ± 0.43	2.46 ± 0.44	0.444
PTV60	3.87 ± 1.55	3.87 ± 1.56	0.912	2.28 ± 0.36	2.30 ± 0.38	0.660	4.32 ± 1.38	4.34 ± 1.39	0.400
PTV54	2.93 ± 0.36	2.91 ± 0.37	0.616	1.85 ± 0.43	1.84 ± 0.42	0.793	3.69 ± 0.48	3.75 ± 0.50	0.020
Brainstem PRV	27.71 ± 7.54	27.27 ± 7.28	0.261	27.72 ± 5.60	29.28 ± 5.99	0.310	27.91 ± 5.86	27.58 ± 6.32	0.498
Bstem	27.48 ± 7.85	27.01 ± 7.58	0.245	27.64 ± 5.70	29.23 ± 6.05	0.285	27.82 ± 6.09	27.51 ± 6.52	0.571
Left eyeballs	6.70 ± 3.40	7.43 ± 3.46	0.927	6.13 ± 2.28	6.50 ± 1.75	0.636	7.76 ± 3.01	8.05 ± 3.61	0.648
Right eyeballs	6.70 ± 2.47	7.83 ± 3.38	0.312	6.39 ± 2.57	6.85 ± 2.01	0.457	7.97 ± 3.65	7.87 ± 3.93	0.674
Larynx	34.23 ± 1.55	34.25 ± 1.53	0.824	35.30 ± 2.36	35.24 ± 2.48	0.925	36.23 ± 3.76	36.28 ± 3.76	0.541
Left lens	4.81 ± 1.48	5.22 ± 1.49	0.452	4.75 ± 1.44	4.93 ± 1.06	0.903	5.47 ± 1.3	5.5 ± 1.41	0.622
Right lens	4.75 ± 1.19	5.27 ± 1.35	0.261	4.70 ± 1.50	5.03 ± 1.33	0.543	5.51 ± 1.39	5.31 ± 1.34	0.648
optic chiasm	31.47 ± 18.64	34.72 ± 18.07	0.729	21.80 ± 16.96	27.96 ± 15.54	0.101	31.23 ± 17.66	31.67 ± 18.85	0.701
Left optic nerves	17.34 ± 10.15	20.82 ± 13.48	0.475	14.02 ± 11.22	16.89 ± 10.65	0.229	22.87 ± 10.9	22.45 ± 12.75	0.701
Right optic nerves	16.84 ± 8.41	19.91 ± 12.17	0.388	14.97 ± 10.25	18.29 ± 10.09	0.181	22.28 ± 12.6	21.5 ± 15.12	0.277
oral cavity	35.18 ± 1.60	35.29 ± 1.97	0.613	36.90 ± 2.63	36.75 ± 2.71	0.456	37.52 ± 3.36	37.23 ± 3.48	0.059
Left parotids	30.37 ± 2.17	30.42 ± 2.42	0.498	32.44 ± 3.44	32.37 ± 3.46	0.839	31.34 ± 3.05	31.04 ± 3.64	0.349
Right parotids	30.52 ± 2.99	30.40 ± 2.87	0.898	31.70 ± 3.15	31.71 ± 2.85	0.839	32.2 ± 5.57	31.94 ± 5.74	0.294
pituitary	45.75 ± 17.20	48.04 ± 17.05	0.216	42.44 ± 16.79	47.13 ± 13.52	0.298	48.72 ± 14.38	48.75 ± 17.02	0.294
Spinal cord PRV	25.26 ± 5.79	24.54 ± 5.29	0.812	26.92 ± 4.74	26.93 ± 3.98	0.818	27.29 ± 3.92	28.65 ± 4.39	0.040*
Spinal cord	25.55 ± 5.90	24.70 ± 5.40	0.729	26.98 ± 5.01	26.76 ± 4.10	0.968	27.23 ± 4.21	28.4 ± 4.6	0.058
Thyroid	28.13 ± 8.21	25.45 ± 9.03	0.070	38.43 ± 7.83	31.90 ± 8.81	<0.001*	38.42 ± 11.9	35.74 ± 7.73	0.083

NTS VMAT, non-thyroid-sparing volume modulated arc therapy; TS VMAT, thyroid-sparing volume modulated arc therapy, *: P<0.05, SD, Standard Deviation.

### 3.5 Penalty score evaluation

A penalty score, derived from a weighted sum of MADD values, was utilized to assess overall plan quality (**Table 6**). The comparative analysis of penalty scores between TS VMAT and NTS VMAT plans across three groups revealed statistically significant differences in two groups. In the Bilateral UNI group (n=20), TS VMAT plans significantly reduced the penalty score in 85% of patients (17/20) compared to NTS VMAT plans. Similarly, for the One-side LNI group (n=20), TS VMAT plans achieved lower penalty scores than NTS VMAT plans in 80% of cases (16/20), with statistically significant differences between the two plans. Conversely, no significant difference was observed in the Bilateral LNI group (n=20), where only 50% of patients (10/20) exhibited reduced penalty scores with TS VMAT plans. These findings suggest that the benefits of TS VMAT optimization are particularly pronounced in upper neck and unilateral lower neck irradiation scenarios.

**Table 6:** The penalty score for three groups in TS VMAT plans and NTS VMAT plans.

Group	NTS VMAT (Mean ± SD)	TS VMAT (Mean ± SD)	P-value
Bilateral upper neck irradiation	21.50 ± 2.87	15.99 ± 4.06	<0.001*
One-side lower neck irradiation	18.12 ± 3.16	14.76 ± 2.8	<0.001*
Bilateral lower neck irradiation	17.81 ± 3.45	17.90 ± 4.01	0.779

NTS VMAT, non-thyroid-sparing volume modulated arc therapy; TS VMAT, thyroid-sparing volume modulated arc therapy, *: P<0.05.

## 4 Discussion

This dosimetric feasibility study encompassed 60 patients diagnosed with non-distant metastatic NPC, categorized into the Bilateral UNI group, the One-side LNI group, and the Bilateral LNI group. These patients were initially treated with standardized VMAT without specific thyroid-sparing techniques. We subsequently re-optimized the original dosimetry plans by designating the thyroid as an OAR and imposing stringent constraints on the radiation dose it received. Our analysis revealed no significant differences in the HI and CI across all PTVs, nor in the dose distributions for OARs between the two plans within the Bilateral UNI group and the One-side LNI group. Conversely, in the Bilateral LNI group, we detected minor variations in the HI and CI for PTVs and in the dose distributions for certain OARs in the TS VMAT plans. Nonetheless, it is important to emphasize that these variations remained within clinically acceptable limits. These findings establish a basis for subsequent investigations into the potential of TS VMAT plans to reduce radiation exposure to the thyroid. Our study demonstrates that, compared to NTS VMAT plans, TS VMAT plans significantly decrease the radiation dose to the thyroid across all groups without worsening the HI, the CI, or increasing irradiation doses to OARs. Notably, even within the Bilateral UNI group and the One-side LNI group of NTS VMAT plans, the thyroid dose meets the established criteria for thyroid-sparing. Nevertheless, TS VMAT plans can further reduce the radiation dose to the thyroid gland, thereby minimizing the risk of hypothyroidism in patients (13). The penalty score analysis confirmed the dosimetric feasibility of TS VMAT, particularly in the Bilateral UNI group and One-side LNI group, where significant improvements were observed. In the bilateral lower neck group, clinically acceptable compromises in spinal cord doses did not outweigh the benefits of thyroid sparing, underscoring the adaptability of TS VMAT across diverse clinical scenarios. This study indicates that HS VMAT plans demonstrate distinct advantages over NHS VMAT plans in terms of thyroid sparing during radiotherapy for patients with non-distant metastatic NPC.

External beam radiotherapy, as a representative deterministic radiation effect, is associated with the onset of hypothyroidism. The likelihood of developing hypothyroidism is significantly correlated with the radiotherapy dose administered. Notably, hypothyroidism seldom manifests following head and neck external beam radiotherapy when the thyroid is exposed to doses below 10 Gy (26). High doses of radiation cause thyroid gland damage, leading to necrosis, apoptosis, and atrophy. Even at lower doses, radiation can induce autoimmune thyroiditis, though the underlying mechanisms are not yet understood (27). It is now believed that acute thyroid dysfunction results from radiation-induced damage to the parenchymal cells of the thyroid gland. In contrast, advanced thyroid dysfunction is attributed to ischemia of the small thyroid vessels and carotid atherosclerosis (28).

Hypothyroidism is the most common toxic reaction to radiotherapy in patients with head and neck tumors, often developing within 5 years, peaking 1–2 years post-treatment. Severe hypothyroidism can adversely impact multiple organ systems, manifesting in circulatory symptoms such as pleural effusion, pericardial effusion, hemodynamic instability, as well as neurological and musculoskeletal symptoms (29). Patients diagnosed with clinical hypothyroidism are typically prescribed levothyroxine, including those with subclinical hypothyroidism who exhibit symptoms indicative of the condition (30, 31). Nevertheless, prolonged administration of levothyroxine in individuals with heart failure may be associated with an increased risk of mortality (32). Furthermore, long-term levothyroxine supplementation necessitates regular monitoring of hormone levels, including thyroxine and TSH, which may impose financial and psychological burdens on patients. Additionally, the incidence of hypothyroidism is significantly associated with the radiotherapy dose. Notably, our study demonstrates that TS VMAT plan is a promising strategy for minimizing radiation exposure to the thyroid gland. It is essential to limit the thyroid gland’s radiation dose to reduce hypothyroidism risk, but thyroid sparing is not advised for patients with tumors nearby, as it may result in treatment failure.

This dosimetric feasibility study has a non-negligible limitation that needs to be considered. Although our simulations of the TS VMAT plans showed impressive benefits, our patient sample size was insufficient to generalize our findings to the general patient population. We didn’t monitor patients’ thyroid function, so we couldn’t confirm if TS VMAT plans reduce hypothyroidism risk. In addition, given the retrospective nature of our study, even though we tried to minimize the occurrence of selection bias, it is possible that there were unaccounted for factors that influenced the inclusion of patients, which in turn led to selection bias. Our encouraging results need to be further validated by more multicenter and prospective clinical trials to establish a clear advantage in the protection of thyroid function in nasopharyngeal carcinoma patients treated with TS VMAT plans. Furthermore, universally accepted standard radiation doses for thyroid sparing have not yet been established, necessitating further research in this area. Despite these limitations, our findings indicate that TS VAMT plans are dosimetrically feasible and capable of reducing radiation exposure to the thyroid.

## 5 Conclusions

The TS VMAT plan demonstrates potential as an effective strategy in radiotherapy planning for patients with non-distant metastatic NPC, primarily due to its ability to lower radiation exposure to the thyroid gland compared to the NTS VMAT plan. This reduction may mitigate the risk of hypothyroidism without worsening the HI, the CI, or increasing irradiation doses to OARs. The promising outcomes of our study warrant further validation through clinical trials with larger sample sizes to definitively establish the benefits of TS VMAT plans in preserving thyroid function in NPC patients.

## Data Availability

The raw data supporting the conclusions of this article will be made available by the authors, without undue reservation.
