# Supplementary material for: Thyroid-sparing volume-modulated arc therapy in patients with non-distant metastatic nasopharyngeal carcinoma: a feasibility study
Source: Front Oncol. 2025 Jun 12;15:1443226. doi: 10.3389/fonc.2025.1443226 (PMC12198196; doi:10.3389/fonc.2025.1443226)
Supplement: Supplementary file 9 [file Table1.docx]

| **Supplementary Table 1.** Dosage distribution in PTVnx, PTVnd, PTV60minus and PTV54minus in NTS VMAT plans and TS VMAT plans in Affiliated Cancer Hospital of Shantou University | | | | | | | | | | |
| --- | --- | --- | --- | --- | --- | --- | --- | --- | --- | --- |
|  | Bilateral upper neck irradiation group | | | One-side lower neck irradiation group | | | Bilateral lower neck irradiation group | | |  |
|  | NTS VMAT | TS VMAT | P-value | NTS VMAT | TS VMAT | P-value | NTS VMAT | TS VMAT | P-value |  |
|  | (Mean±SD) | (Mean±SD) |  | (Mean±SD) | (Mean±SD) |  | (Mean±SD) | (Mean±SD) |  |  |
| PTVnx |  |  |  |  |  |  |  |  |  |  |
| D98 (Gy) | 69.88±0.34 | 69.88±0.34 | 0.772 | 69.95±0.42 | 69.82±0.57 | 0.093 | 70.06±0.43 | 70.06±0.43 | 0.664 |  |
| D50 (Gy) | 72.45±0.24 | 72.42±0.22 | 0.338 | 72.59±0.26 | 72.61±0.28 | 0.539 | 72.63±0.22 | 72.63±0.23 | 0.976 |  |
| D2 (Gy) | 73.48±0.35 | 73.60±0.48 | 0.285 | 73.70±0.42 | 73.76±0.41 | 0.151 | 73.73±0.28 | 73.77±0.35 | 0.467 |  |
| HI | 0.05±0.01 | 0.05±0.01 | 0.333 | 0.05±0.01 | 0.05±0.01 | 0.047* | 0.05±0.01 | 0.05±0.01 | 0.438 |  |
| CI | 0.84±0.03 | 0.84±0.02 | 0.168 | 0.66±0.16 | 0.65±0.16 | 0.385 | 0.56±0.18 | 0.56±0.17 | 0.847 |  |
| PTVnd |  |  |  |  |  |  |  |  |  |  |
| D98 (Gy) | - | - | - | 67.82±0.39 | 67.80±0.39 | 0.734 | 67.58±0.25 | 67.51±0.21 | 0.089 |  |
| D50 (Gy) | - | - | - | 70.25±0.28 | 70.26±0.32 | 0.949 | 70.33±0.30 | 70.31±0.26 | 0.790 |  |
| D2 (Gy) | - | - | - | 71.58±0.44 | 71.62±0.67 | 0.773 | 71.89±0.53 | 71.87±0.52 | 0.858 |  |
| HI | - | - | - | 0.05±0.01 | 0.05±0.01 | 0.618 | 0.06±0.01 | 0.06±0.02 | 0.410 |  |
| CI | - | - | - | 0.27±0.16 | 0.27±0.16 | 0.359 | 0.36±0.15 | 0.36±0.15 | 0.858 |  |
| PTV60minus |  |  |  |  |  |  |  |  |  |  |
| D98 (Gy) | 60.01±0.94 | 60.05±0.95 | 0.684 | 60.46±0.64 | 60.23±0.80 | 0.238 | 60.49±0.71 | 60.44±0.88 | 0.823 |  |
| D50 (Gy) | 65.41±0.36 | 65.43±0.37 | 0.699 | 65.40±0.80 | 65.33±0.84 | 0.346 | 65.58±0.77 | 65.56±0.75 | 0.649 |  |
| D2 (Gy) | 69.51±0.80 | 69.45±0.85 | 0.649 | 69.50±0.90 | 69.47±0.86 | 0.855 | 70.03±0.60 | 70.27±0.69 | 0.079 |  |
| HI | 0.15±0.02 | 0.14±0.02 | 0.516 | 0.14±0.02 | 0.14±0.01 | 0.344 | 0.15±0.01 | 0.15±0.01 | 0.154 |  |
| PTV60 |  |  |  |  |  |  |  |  |  |  |
| CI | 0.70±0.06 | 0.71±0.06 | 0.231 | 0.46±0.15 | 0.46±0.15 | 0.996 | 0.36±0.15 | 0.36±0.15 | 0.872 |  |
| PTV54minus |  |  |  |  |  |  |  |  |  |  |
| D98 (Gy) | 52.97±0.24 | 52.97±0.24 | 0.988 | 53.03±0.14 | 53.00±0.19 | 0.601 | 52.93±0.29 | 52.79±0.38 | 0.006* |  |
| D50 (Gy) | 56.87±0.33 | 56.86±0.25 | 0.674 | 57.03±0.22 | 57.06±0.19 | 0.283 | 57.17±0.32 | 57.19±0.32 | 0.479 |  |
| D2 (Gy) | 59.79±0.78 | 59.77±0.79 | 0.646 | 63.00±1.00 | 63.01±1.08 | 0.830 | 64.46±0.75 | 64.41±0.60 | 0.418 |  |
| HI | 0.12±0.02 | 0.12±0.02 | 0.803 | 0.17±0.02 | 0.18±0.02 | 0.649 | 0.20±0.01 | 0.20±0.01 | 0.371 |  |
| PTV54 |  |  |  |  |  |  |  |  |  |  |
| CI | 0.91±0.01 | 0.91±0.01 | 0.757 | 0.90±0.01 | 0.90±0.01 | 0.066 | 0.89±0.01 | 0.89±0.01 | 0.226 |  |

NTS VMAT: non-thyroid-sparing volume-modulated arc therapy, TS VMAT: thyroid-sparing volume-modulated arc therapy, PTVnx: Planning Target Volume of nasopharynx, PTVnd: Planning Target Volume of the metastatic lymph nodes, PTV60: Planning Target Volume receiving 60 Gy, PTV60minus: PTV60 minus PTVnx and PTVnd 3 mm expansion volume, PTV54: Planning Target Volume receiving 54 Gy, PTV54minus: PTV54 minus PTV60, PTVnx and PTVnd 3 mm expansion volume, D98: dose to 98% volume, D50: dose to 50% volume, D2: dose to 2% volume, HI: homogeneity index, CI: conformity index, *: P<0.05, SD: Standard Deviation
