# Supplementary material for: Thyroid-sparing volume-modulated arc therapy in patients with non-distant metastatic nasopharyngeal carcinoma: a feasibility study
Source: Front Oncol. 2025 Jun 12;15:1443226. doi: 10.3389/fonc.2025.1443226 (PMC12198196; doi:10.3389/fonc.2025.1443226)
Supplement: Supplementary file 10 [file Table2.docx]

| **Supplementary Table 2**. Dosage distribution in OARs in NTS VMAT plans and TS VMAT plans in  Affiliated Cancer Hospital of Shantou University | | | | | | | | | |
| --- | --- | --- | --- | --- | --- | --- | --- | --- | --- |
|  | Bilateral upper neck irradiation group | | | One-side lower neck irradiation group | | | Bilateral lower neck irradiation group | | |
|  | NTS VMAT | TS VMAT | P-value | NTS VMAT | TS VMAT | P-value | NTS VMAT | TS VMAT | P-value |
|  | (Mean±SD) | (Mean±SD) |  | (Mean±SD) | (Mean±SD) |  | (Mean±SD) | (Mean±SD) |  |
| Brainstem PRV | | | | | | | | | |
| Dmax (Gy) | 54.85±6.30 | 54.62±6.58 | 0.312 | 57.38±4.09 | 57.33±4.15 | 0.781 | 55.89±5.15 | 55.69±5.35 | 0.175 |
| Brainstem |  |  |  |  |  |  |  |  |  |
| Dmax (Gy) | 45.42±6.93 | 45.40±6.60 | 0.905 | 47.71±6.05 | 47.64±6.06 | 0.780 | 47.02±6.41 | 47.10±6.29 | 0.717 |
| Spinal cord PRV | | | | | | | | | |
| Dmax (Gy) | 46.74±3.69 | 46.64±3.82 | 0.721 | 46.91±3.01 | 47.19±2.37 | 0.878 | 47.32±2.29 | 47.55±2.88 | 0.139 |
| Spinal cord |  |  |  |  |  |  |  |  |  |
| Dmax (Gy) | 37.81±2.59 | 38.35±2.80 | 0.122 | 38.50±1.51 | 38.20±1.43 | 0.180 | 39.12±2.30 | 39.79±2.66 | 0.028* |
| Left lens |  |  |  |  |  |  |  |  |  |
| Dmax (Gy) | 6.31±1.58 | 6.18±1.49 | 0.288 | 6.09±1.41 | 6.11±1.38 | 0.844 | 7.23±1.01 | 7.49±1.43 | 0.200 |
| Right lens |  |  |  |  |  |  |  |  |  |
| Dmax (Gy) | 6.21±1.50 | 6.15±1.37 | 0.456 | 6.22±1.85 | 6.12±1.74 | 0.446 | 7.11±1.37 | 7.13±1.56 | 0.881 |
| Left optic nerves | | | | | | | | | |
| Dmax (Gy) | 32.22±21.67 | 32.39±21.59 | 0.721 | 21.40±15.79 | 21.61±15.94 | 0.333 | 39.16±18.40 | 39.71±18.43 | 0.096 |
| Right optic nerves | | | | | | | | | |
| Dmax (Gy) | 28.58±17.92 | 28.14±17.60 | 0.254 | 26.53±16.80 | 26.02±16.82 | 0.330 | 38.08±18.12 | 38.73±18.11 | 0.131 |
| optic chiasm |  |  |  |  |  |  |  |  |  |
| Dmax (Gy) | 31.96±22.05 | 31.87±21.94 | 0.241 | 27.24±18.06 | 26.40±18.54 | 0.445 | 38.46±20.84 | 36.84±22.33 | 0.285 |
| Left parotids |  |  |  |  |  |  |  |  |  |
| Dmean (Gy) | 30.63±3.07 | 30.41±3.08 | 0.025* | 34.51±3.66 | 34.45±3.64 | 0.340 | 32.50±3.99 | 32.38±4.19 | 0.221 |
| Right parotids | | | | | | | | | |
| Dmean (Gy) | 30.75±3.58 | 30.69±3.58 | 0.673 | 33.18±3.16 | 33.25±3.03 | 0.536 | 34.31±7.39 | 34.36±7.44 | 0.594 |
| oral cavity |  |  |  |  |  |  |  |  |  |
| Dmean (Gy) | 34.13±1.62 | 34.06±1.89 | 0.739 | 37.85±3.40 | 37.59±3.70 | 0.318 | 39.10±4.24 | 39.01±4.24 | 0.503 |
| larynx |  |  |  |  |  |  |  |  |  |
| Dmean (Gy) | 35.53±1.76 | 35.44±1.83 | 0.139 | 37.29±1.69 | 37.28±1.91 | 0.878 | 39.21±3.18 | 39.28±3.15 | 0.594 |
| Left eyeballs |  |  |  |  |  |  |  |  |  |
| Dmax (Gy) | 23.63±9.66 | 21.78±9.05 | 0.006* | 21.35±6.27 | 20.35±5.85 | 0.159 | 25.89±7.00 | 25.89±7.11 | 0.998 |
| Right eyeballs | | | | | | | | | |
| Dmax (Gy) | 22.50±7.45 | 22.49±7.49 | 0.995 | 20.91±6.24 | 20.41±5.86 | 0.468 | 27.40±14.10 | 27.46±14.11 | 0.931 |
| pituitary |  |  |  |  |  |  |  |  |  |
| Dmean (Gy) | 38.35±19.62 | 38.58±19.18 | 0.395 | 42.42±17.50 | 42.52±17.27 | 0.675 | 45.43±20.06 | 46.84±19.66 | 0.386 |

NTS VMAT: non-thyroid-sparing volume-modulated arc therapy, TS VMAT: thyroid-sparing volume-modulated arc therapy, PRV: planning organs-at-risk volume, Dmax: maximum dose, Dmean: mean dose, *: P<0.05, SD: Standard Deviation
