# Supplementary material for: Thyroid-sparing volume-modulated arc therapy in patients with non-distant metastatic nasopharyngeal carcinoma: a feasibility study
Source: Front Oncol. 2025 Jun 12;15:1443226. doi: 10.3389/fonc.2025.1443226 (PMC12198196; doi:10.3389/fonc.2025.1443226)
Supplement: Supplementary file 11 [file Table3.docx]

| **Supplementary Table 3.** Dosage distribution in the thyroid in NTS VMAT plans and TS VMAT plans  in Affiliated Cancer Hospital of Shantou University | | | | | | | | | |
| --- | --- | --- | --- | --- | --- | --- | --- | --- | --- |
|  | Bilateral upper neck irradiation group | | | One-side lower neck irradiation group | | | Bilateral lower neck irradiation group | | |
|  | NTS VMAT | TS VMAT | P-value | NTS VMAT | TS VMAT | P-value | NTS VMAT | TS VMAT | P-value |
|  | (Mean±SD) | (Mean±SD) |  | (Mean±SD) | (Mean±SD) |  | (Mean±SD) | (Mean±SD) |  |
| Thyroid |  |  |  |  |  |  |  |  |  |
| Dmin | 4.20±1.28 | 3.83±0.86 | 0.005* | 16.44±6.97 | 6.75±2.02 | 0.000* | 29.56±8.68 | 11.62±2.44 | 0.000* |
| (Gy) |  |  |  |  |  |  |  |  |  |
| Dmean (Gy) | 25.73±5.00 | 20.52±3.00 | 0.000* | 38.50±4.35 | 27.87±5.84 | 0.000* | 47.04±3.55 | 39.03±4.45 | 0.005* |
| Dmax (Gy) | 58.06±1.02 | 58.22±0.86 | 0.605 | 59.14±2.13 | 59.66±2.21 | 0.046* | 60.66±3.92 | 60.33±3.92 | 0.285 |
| V40 | 27.93±8.12 | 21.09±5.51 | 0.003* | 49.26±14.32 | 33.46±12.87 | 0.000* | 79.70±16.21 | 53.94±13.47 | 0.000* |

NTS VMAT: non-thyroid-sparing volume-modulated arc therapy, TS VMAT: thyroid-sparing volume-modulated arc therapy, Dmax: maximum dose, Dmean: mean dose, Dmin: minimum dose, V40: the volume irradiated with 40 Gy or more, *: P<0.05, SD: Standard Deviation
