# Supplementary material for: Thyroid-sparing volume-modulated arc therapy in patients with non-distant metastatic nasopharyngeal carcinoma: a feasibility study
Source: Front Oncol. 2025 Jun 12;15:1443226. doi: 10.3389/fonc.2025.1443226 (PMC12198196; doi:10.3389/fonc.2025.1443226)
Supplement: Supplementary file 12 [file Table4.docx]

| **Supplementary Table 4**. Dosage distribution in PTVnx, PTVnd, PTV60_minus_ and PTV54_minus_ in NTS VMAT plans  and TS VMAT plans in Jieyang People’s Hospital | | | | | | | | | |
| --- | --- | --- | --- | --- | --- | --- | --- | --- | --- |
|  | Bilateral upper neck irradiation group | | | One-side lower neck irradiation group | | | Bilateral lower neck irradiation group | | |
|  | NTS VMAT | TS VMAT | P-value | NTS VMAT | TS VMAT | P-value | NTS VMAT | TS VMAT | P-value |
|  | (Mean±SD) | (Mean±SD) |  | (Mean±SD) | (Mean±SD) |  | (Mean±SD) | (Mean±SD) |  |
| PTVnx |  |  |  |  |  |  |  |  |  |
| D98 (Gy) | 69.95±0.14 | 69.96±0.15 | 0.544 | 69.81±0.20 | 69.83±0.19 | 0.449 | 69.81±0.20 | 69.82±0.22 | 0.489 |
| D50 (Gy) | 72.20±0.33 | 72.15±0.36 | 0.319 | 72.06±0.44 | 72.05±0.44 | 0.587 | 72.43±0.42 | 72.44±0.46 | 0.648 |
| D2 (Gy) | 73.48±0.47 | 73.42±0.50 | 0.391 | 73.46±0.72 | 73.42±0.66 | 0.534 | 73.94±0.59 | 73.98±0.60 | 0.380 |
| HI | 0.05±0.01 | 0.05±0.01 | 0.387 | 0.05±0.01 | 0.05±0.01 | 0.221 | 0.06±0.01 | 0.06±0.01 | 0.575 |
| CI | 0.90±0.02 | 0.90±0.02 | 0.464 | 0.66±0.18 | 0.66±0.19 | 0.569 | 0.56±0.12 | 0.56±0.13 | 0.896 |
| PTVnd |  |  |  |  |  |  |  |  |  |
| D98 (Gy) | - | - | - | 67.87±1.01 | 68.15±0.46 | 0.959 | 67.85±0.37 | 67.81±0.41 | 0.154 |
| D50 (Gy) | - | - | - | 70.53±0.48 | 70.50±0.39 | 0.501 | 70.97±1.01 | 70.80±0.48 | 0.878 |
| D2 (Gy) | - | - | - | 72.25±0.54 | 72.16±0.54 | 0.014* | 72.85±0.69 | 72.97±0.74 | 0.059 |
| HI | - | - | - | 0.06±0.02 | 0.06±0.01 | 0.114 | 0.07±0.01 | 0.07±0.01 | 0.01* |
| CI | - | - | - | 0.24±0.19 | 0.24±0.19 | 0.119 | 0.36±0.11 | 0.36±0.11 | 0.053 |
| PTV60minus |  |  |  |  |  |  |  |  |  |
| D98 (Gy) | 60.53±0.37 | 60.52±0.43 | 0.966 | 60.07±0.58 | 60.11±0.48 | 0.263 | 60.35±0.28 | 60.35±0.31 | 0.789 |
| D50 (Gy) | 64.05±1.85 | 64.00±1.84 | 0.317 | 63.44±0.96 | 63.40±0.91 | 0.250 | 62.93±0.64 | 62.97±0.70 | 0.432 |
| D2 (Gy) | 68.16±2.44 | 68.09±2.42 | 0.252 | 67.93±1.77 | 67.86±1.69 | 0.143 | 66.38±0.68 | 66.42±0.71 | 0.431 |
| HI | 0.12±0.03 | 0.12±0.03 | 0.177 | 0.12±0.03 | 0.12±0.03 | 0.063 | 0.10±0.01 | 0.10±0.01 | 0.447 |
| PTV60 |  |  |  |  |  |  |  |  |  |
| CI | 0.81±0.08 | 0.81±0.08 | 0.878 | 0.57±0.12 | 0.57±0.12 | 0.601 | 0.53±0.10 | 0.51±0.10 | 0.001* |
| PTV54minus |  |  |  |  |  |  |  |  |  |
| D98 (Gy) | 52.63±0.81 | 52.40±1.10 | 0.152 | 52.64±0.83 | 52.42±1.05 | 0.203 | 52.59±0.68 | 51.40±0.70 | 0.000* |
| D50 (Gy) | 57.18±0.36 | 57.16±0.42 | 0.308 | 57.21±0.36 | 57.18±0.37 | 0.377 | 57.65±0.54 | 57.75±0.60 | 0.017* |
| D2 (Gy) | 59.58±1.02 | 59.66±1.09 | 0.799 | 63.48±1.40 | 63.24±1.44 | 0.063 | 63.50±1.31 | 63.69±1.23 | 0.072 |
| HI | 0.12±0.03 | 0.13±0.03 | 0.151 | 0.19±0.03 | 0.19±0.03 | 0.934 | 0.19±0.03 | 0.21±0.03 | 0.000* |
| PTV54 |  |  |  |  |  |  |  |  |  |
| CI | 0.87±0.02 | 0.87±0.02 | 0.668 | 0.84±0.02 | 0.85±0.02 | 0.799 | 0.84±0.02 | 0.83±0.02 | 0.003* |

NTS VMAT: non-thyroid-sparing volume-modulated arc therapy, TS VMAT: thyroid-sparing volume-modulated arc therapy, PTVnx: Planning Target Volume of nasopharynx, PTVnd: Planning Target Volume of the metastatic lymph nodes, PTV60: Planning Target Volume receiving 60 Gy, PTV60_minus_: PTV60 minus PTVnx and PTVnd 3 mm expansion volume, PTV54: Planning Target Volume receiving 54 Gy, PTV54_minus_: PTV54 minus PTV60, PTVnx and PTVnd 3 mm expansion volume, D98: dose to 98% volume, D50: dose to 50% volume, D2: dose to 2% volume, HI: homogeneity index, CI: conformity index, *: P<0.05, SD: Standard Deviation
