# Supplementary material for: Thyroid-sparing volume-modulated arc therapy in patients with non-distant metastatic nasopharyngeal carcinoma: a feasibility study
Source: Front Oncol. 2025 Jun 12;15:1443226. doi: 10.3389/fonc.2025.1443226 (PMC12198196; doi:10.3389/fonc.2025.1443226)
Supplement: Supplementary file 13 [file Table5.docx]

| **Supplementary Table 5**. Dosage distribution in OARs in NTS VMAT plans and TS VMAT plans  in Jieyang People’s Hospital | | | | | | | | | |
| --- | --- | --- | --- | --- | --- | --- | --- | --- | --- |
|  | Bilateral upper neck irradiation group | | | One-side lower neck irradiation group | | | Bilateral lower neck irradiation group | | |
|  | NTS VMAT | TS VMAT | P-value | NTS VMAT | TS VMAT | P-value | NTS VMAT | TS VMAT | P-value |
|  | (Mean±SD) | (Mean±SD) |  | (Mean±SD) | (Mean±SD) |  | (Mean±SD) | (Mean±SD) |  |
| Brainstem PRV | | | | | | | | | |
| Dmax (Gy) | 58.61±2.68 | 58.53±2.77 | 0.630 | 58.84±2.01 | 58.69±1.77 | 0.464 | 58.62±2.40 | 58.58±2.47 | 0.079 |
| Brainstem |  |  |  |  |  |  |  |  |  |
| Dmax (Gy) | 52.68±2.76 | 52.64±2.91 | 0.821 | 52.37±0.85 | 52.71±1.82 | 0.646 | 52.41±1.31 | 52.13±1.46 | 0.079 |
| Spinal cord PRV | | | | | | | | | |
| Dmax (Gy) | 46.21±4.08 | 45.43±1.55 | 0.959 | 45.52±1.42 | 45.73±1.02 | 0.456 | 46.87±1.91 | 46.97±2.12 | 0.503 |
| Spinal cord |  |  |  |  |  |  |  |  |  |
| Dmax (Gy) | 40.16±1.21 | 40.08±1.18 | 0.322 | 40.34±0.97 | 40.43±1.09 | 0.605 | 40.91±0.91 | 41.36±0.96 | 0.004* |
| Left lens |  |  |  |  |  |  |  |  |  |
| Dmax (Gy) | 6.07±1.51 | 5.95±1.55 | 0.125 | 5.33±1.64 | 5.43±1.60 | 0.311 | 5.73±1.47 | 5.76±1.57 | 0.646 |
| Right lens |  |  |  |  |  |  |  |  |  |
| Dmax (Gy) | 6.00±1.59 | 5.97±1.64 | 0.594 | 5.34±1.65 | 5.41±1.70 | 0.467 | 5.62±1.49 | 5.64±1.51 | 0.600 |
| Left optic nerves | | | | | | | | | |
| Dmax (Gy) | 42.26±10.93 | 42.04±3.14 | 0.333 | 35.23±17.78 | 36.06±17.30 | 0.402 | 36.56±18.29 | 36.88±18.26 | 0.100 |
| Right optic nerves | | | | | | | | | |
| Dmax (Gy) | 39.22±10.30 | 38.91±9.64 | 0.606 | 33.05±15.12 | 33.16±15.29 | 0.886 | 36.17±16.47 | 36.05±16.19 | 0.732 |
| optic chiasm |  |  |  |  |  |  |  |  |  |
| Dmax (Gy) | 42.26±13.15 | 41.90±13.40 | 0.408 | 41.61±16.19 | 42.10±15.13 | 0.413 | 39.07±17.71 | 38.46±17.86 | 0.386 |
| Left parotids |  |  |  |  |  |  |  |  |  |
| Dmean (Gy) | 29.85±1.12 | 29.87±1.07 | 0.421 | 29.94±2.02 | 29.89±2.01 | 0.241 | 27.50±9.62 | 30.82±1.36 | 0.047* |
| Right parotids | | | | | | | | | |
| Dmean (Gy) | 29.76±1.45 | 29.74±1.42 | 0.496 | 29.37±1.81 | 29.36±1.73 | 0.851 | 31.00±1.96 | 31.20±2.01 | 0.005* |
| oral cavity |  |  |  |  |  |  |  |  |  |
| Dmean (Gy) | 33.21±1.55 | 33.23±1.54 | 0.789 | 33.10±1.19 | 33.16±0.99 | 0.878 | 33.69±1.21 | 33.78±1.21 | 0.017* |
| larynx |  |  |  |  |  |  |  |  |  |
| Dmean (Gy) | 33.20±0.34 | 33.16±0.34 | 0.112 | 33.31±0.29 | 33.26±0.27 | 0.017* | 33.38±0.26 | 33.36±0.22 | 0.335 |
| Left eyeballs |  |  |  |  |  |  |  |  |  |
| Dmax (Gy) | 13.50±4.33 | 13.60±4.25 | 0.445 | 13.28±5.36 | 14.25±6.68 | 0.013* | 14.65±7.64 | 14.69±7.70 | 0.796 |
| Right eyeballs | | | | | | | | | |
| Dmax (Gy) | 14.67±4.10 | 14.84±3.77 | 0.696 | 16.98±10.68 | 17.35±10.93 | 0.199 | 16.09±7.74 | 16.21±7.92 | 0.576 |
| pituitary |  |  |  |  |  |  |  |  |  |
| Dmean (Gy) | 51.50±6.17 | 51.82±5.98 | 0.404 | 51.23±12.63 | 53.22±10.88 | 0.074 | 49.70±15.64 | 48.90±16.30 | 0.575 |

NTS VMAT: non-thyroid-sparing volume-modulated arc therapy, TS VMAT: thyroid-sparing volume-modulated arc therapy, PRV: planning organs-at-risk volume, Dmax: maximum dose, Dmean: mean dose, *: P<0.05, SD: Standard Deviation
