# Supplementary material for: Thyroid-sparing volume-modulated arc therapy in patients with non-distant metastatic nasopharyngeal carcinoma: a feasibility study
Source: Front Oncol. 2025 Jun 12;15:1443226. doi: 10.3389/fonc.2025.1443226 (PMC12198196; doi:10.3389/fonc.2025.1443226)
Supplement: Supplementary file 14 [file Table6.docx]

| **Supplementary Table 6**. Dosage distribution in the thyroid in NTS VMAT plans and TS VMAT plans  in Jieyang People’s Hospital | | | | | | | | | |
| --- | --- | --- | --- | --- | --- | --- | --- | --- | --- |
|  | Bilateral upper neck irradiation group | | | One-side lower neck irradiation group | | | Bilateral lower neck irradiation group | | |
|  | NTS VMAT | TS VMAT | P-value | NTS VMAT | TS VMAT | P-value | NTS VMAT | TS VMAT | P-value |
|  | (Mean±SD) | (Mean±SD) |  | (Mean±SD) | (Mean±SD) |  | (Mean±SD) | (Mean±SD) |  |
| Thyroid |  |  |  |  |  |  |  |  |  |
| Dmin | 6.32±2.25 | 4.46±1.55 | 0.005* | 21.84±7.71 | 8.56±1.91 | 0.000* | 30.18±3.09 | 12.82±1.97 | 0.000* |
| (Gy) |  |  |  |  |  |  |  |  |  |
| Dmean (Gy) | 33.60±7.06 | 20.59±5.17 | 0.005* | 41.22±4.11 | 24.48±2.66 | 0.000* | 47.66±1.07 | 29.32±2.54 | 0.000* |
| Dmax (Gy) | 57.80±1.61 | 55.83±3.22 | 0.013* | 59.79±4.13 | 59.81±4.86 | 0.959 | 59.96±3.80 | 57.93±5.13 | 0.059 |
| V40 | 75.49±97.81 | 14.90±7.09 | 0.005* | 58.50±12.58 | 15.96±7.94 | 0.000* | 91.36±3.91 | 21.54±6.25 | 0.000* |

NTS VMAT: non-thyroid-sparing volume-modulated arc therapy, TS VMAT: thyroid-sparing volume-modulated arc therapy, Dmax: maximum dose, Dmean: mean dose, Dmin: minimum dose, V40: the volume irradiated with 40 Gy or more, *: P<0.05, SD: Standard Deviation
